# Zap70 Regulates TCR-Mediated Zip6 Activation at the Immunological Synapse

**DOI:** 10.3389/fimmu.2021.687367

**Published:** 2021-07-29

**Authors:** Bonah Kim, Hee Young Kim, Won-Woo Lee

**Affiliations:** ^1^Laboratory of Autoimmunity and Inflammation (LAI), Department of Biomedical Sciences, BK21Plus Biomedical Science Project, Seoul National University College of Medicine, Seoul, South Korea; ^2^Department of Microbiology and Immunology, Seoul National University College of Medicine, Seoul, South Korea; ^3^Institute of Infectious Diseases, Seoul National University College of Medicine, Seoul, South Korea; ^4^Cancer Research Institute and Ischemic/Hypoxic Disease Institute, Seoul National University College of Medicine, Seoul National University Hospital Biomedical Research Institute, Seoul, South Korea

**Keywords:** zinc, Zip6, T lymphocytes, lipid rafts, immunological synapse, T cell receptor (TCR)

## Abstract

The essential microelement zinc plays immunoregulatory roles *via* its ability to influence signaling pathways. Zinc deficiency impairs overall immune function and resultantly increases susceptibility to infection. Thus, zinc is considered as an immune-boosting supplement for populations with hypozincemia at high-risk for infection. Besides its role as a structural cofactor of many proteins, zinc also acts as an intracellular messenger in immune cell signaling. T-cell activation instructs zinc influx from extracellular and subcellular sources through the Zip6 and Zip8 zinc transporters, respectively. Increased cytoplasmic zinc participates in the regulation of T-cell responses by modifying activation signaling. However, the mechanism underlying the activation-dependent movement of zinc ions by Zip transporters in T cells remains elusive. Here, we demonstrate that Zip6, one of the most abundantly expressed Zip transporters in T cells, is mainly localized to lipid rafts in human T cells and is recruited into the immunological synapse in response to TCR stimulation. This was demonstrated through confocal imaging of the interaction between CD4^+^ T cells and antigen-presenting cells. Further, immunoprecipitation assays show that TCR triggering induces tyrosine phosphorylation of Zip6, which has at least three putative tyrosine motifs in its long cytoplasmic region, and this phosphorylation is coupled with its physical interaction with Zap70. Silencing Zip6 reduces zinc influx from extracellular sources and suppresses T-cell responses, suggesting an interaction between Zip6-mediated zinc influx and TCR activation. These results provide new insights into the mechanism through which Zip6-mediated zinc influx occurs in a TCR activation-dependent manner in human CD4^+^ T cells.

## Introduction

Zinc is one of the most important trace elements in the body and functions as an important cofactor in multiple biological processes. This ion not only plays structural and catalytic roles in regulating thousands of metalloproteins (1, 2), a large body of evidence indicates that zinc also acts as an intracellular ionic signaling molecule contributing to the regulation of signaling pathways. Dysregulation of zinc concentration in the cytoplasm could cause severe impairment of cellular integrity and survival. Thus, intracellular zinc homeostasis is tightly maintained through spatiotemporal and elaborate regulation of zinc-specific transporters and binding proteins (3). Two families of proteins, the solute-linked carrier 39 (SLC39A, or Zip) family of zinc importers and solute-linked carrier 30 family (SLC30A, or ZnT) of zinc exporters, coordinately guide movement of zinc ions across the plasma membrane. Zinc transporters are expressed in a cell- or tissue-specific manner and their dysfunction is broadly associated with pathogenesis of many diseases (4).

It has been demonstrated that zinc contributes to the growth, development and integrity of the immune system, as well as to immune responses (5, 6). Zinc deficiency adversely affects both innate and adaptive immunity, as its deficiency in innate immune cells leads to reduced chemotaxis of polymorphonuclear cells (PMNs), phagocytosis of macrophages, and diminished ROS production (5). In the case of adaptive immunity, zinc deficiency causes T- and B-cell lymphopenia, imbalance among the different helper T cell subsets, and defective antibody production (7–9). In addition, cytokine production is markedly influenced by zinc deficiency in both innate and adaptive immune cells (10, 11). Clinically, zinc deficiency caused by malnutrition and dyshomeostasis results in increased host susceptibility to various infections (12). Thus, zinc supplementation is considered to be beneficial for recovering immune function in many bacterial and viral infectious diseases.

Similar to what happens with calcium ions, immune cells generally increase their intracellular zinc content during activation (13, 14). Accumulation of cytosolic zinc can be accomplished through increased zinc influx mediated by Zip transporters and/or downregulation of ZnT-induced zinc efflux in activated immune cells. Recent studies have highlighted important roles of Zip7, Zip8, and Zip10 in immune cells. Zip7 and Zip10 play an essential role for B cell development and BCR-induced B cell proliferation in mice, whereas Zip8-mediated zinc influx regulates the NF-κB pathway in monocytes/macrophages and elicits IFN-γ expression in activated T cells (7, 15). In particular, previous reports by our group and others have revealed that Zip6, which is mainly expressed on the plasma membrane of T cells, is responsible for zinc influx from extracellular sources during T-cell activation. Intracellular zinc concentrations increase rapidly after T cell receptor (TCR) triggering, in particular in the subsynaptic area of the T cell–dendritic cell (DC) interaction platform. Increased zinc influx strengthened early events of TCR signaling as a result of the inhibition of the SHP-1 recruitment and increased ZAP70 phosphorylation (16). In addition, Zip6 is constitutively expressed in human and murine T cells and its expression is upregulated upon TCR stimulation. Studies in which Zip6 deficiency is achieved *via* siRNA or CRISPR/Cas9 show that loss of this transporter results in impaired T cell activation. Thus, Zip6 is considered a critical component of the T cell activation machinery (17). Despite their importance for regulating cytoplasmic zinc homeostasis in T cells, the mechanisms underlying how zinc transporters are activated to move zinc ions across the cell membrane is still poorly understood.

Mechanisms of zinc transport have been recently proposed based on crystal structures of prokaryotic zinc transporters, such as YiiP from *Escherichia coli* and BbZIP from *Bordetella bronchiseptica*, that show homology with mammalian ZnTs and human Zip4, respectively (18, 19). In addition, the model of BbZIP suggests that, unlike typical ion channels, a significant conformational change is required for zinc transport with this channel (19). More recently, it has been suggested that exogenous stimulation results in activation of Zip7 through its phosphorylation by the protein kinase CK2, which leads to the release of ER-stored zinc into cytoplasm (20). Based on previous findings by ourselves and others, we hypothesized that activation-induced zinc influx is mediated by accumulation of Zip6 into the immunological synapse (IS) and by phosphorylation of Zip6 early in TCR signal transduction.

Here, we provide evidence that Zip6 is preferentially localized to lipid rafts and accumulated to the immunological synapse upon TCR stimulation. Zip6 activation was found to be closely associated with proximal TCR signaling and is regulated by Zap70-mediated phosphorylation during TCR stimulation. Moreover, the increase in cytoplasmic zinc caused by Zip6 is important for activation and function of CD4^+^ T cells in humans. Our data provide new insights into how the activation of Zip6, which accounts for zinc influx in human T cells, is regulated by TCR stimulation.

## Materials and Methods

### Cell Preparation

The study protocols were approved by the institutional review board of Seoul National University Hospital. Peripheral blood of healthy donors was drawn after obtaining written, informed consent. The methods were performed in accordance with the approved guidelines. Peripheral blood mononuclear cells (PBMCs) were isolated from blood by density gradient centrifugation (Biocoll separating solution; BIOCHROM Inc., Cambridge, UK). Total CD4^+^ T cells were negatively separated from CD14^-^ cells with MojoSort™ Human total CD4^+^ T Cell Isolation Kit according to the manufacturer’s instructions (BioLegend, San Diego, CA, USA). For *in vitro* activation, T cells (1 × 10^6^/ml) were incubated with anti-CD3 (1.5 μg/ml) and anti-CD28 (1 μg/ml) antibodies (Abs) on ice, followed by cross-linking with goat-anti-mouse IgG (1.5 μg/ml) at 37°C.

### Antibodies and Reagents

Anti-Zip6 Abs were obtained from Novus Biologicals (Centennial, CO, USA) and Abcam (Cambridge, UK). In addition, human anti-Zip6 polyclonal antiserum was developed by GW Viteck (Seoul, Republic of Korea) for immunoprecipitation. Anti-CD3 and Flotilin-1 Abs were purchased from BD Biosciences (San Jose, CA, USA), Anti-Lck Ab was obtained from Santacruz (Dallas, TX, USA). Anti-CD71 and Zap70 Abs were purchased from Cell Signaling Technology (Danvers, MA, USA). Cholera Toxin Subunit B (Recombinant), Horseradish Peroxidase Conjugate was obtained from Invitrogen (Waltham, MA, USA) and anti-β-actin Ab was obtained from MilliporeSigma (Burlington, MA, USA), respectively. SEE (Staphylococcal enterotoxin E), SEB (Staphylococcal enterotoxin B), and TSST-1 (Toxic shock syndrome toxin 1) were purchased from Toxin Technology Inc. (Sarasota, FL, USA) according to the regulations, aliquoted in small amounts and stored at -80°C until use. Lck inhibitor (RK-24466) and Zap70 inhibitor (Zap 180013) were obtained from Cayman Chemical (Ann Arbor, MI, USA) and TOCRIS (Ellisville, MO, USA), respectively.

### Sucrose Gradient Centrifugation

Cells (2.5 × 10^7^) were washed twice with PBS and lysed in 2 ml ice-cold sodium carbonate buffer containing 500 mM sodium carbonate, 25 mM MES and 150 mM NaCl, 1% Triton-X 100 and protease inhibitors, and homogenized using a loose-fitting Dounce homogenizer (40 strokes). The lysate was adjusted to 40% sucrose by the addition of an equal volume of 80% sucrose and placed at the bottom of an ultracentrifuge tube (Beckman Instruments, Fullerton, CA, USA). A 5% and 35% discontinuous sucrose gradient (4 ml 5% sucrose and 4 ml 35% sucrose, both in 25 mM MES buffer) was formed above the sample and centrifuged at 38,000 rpm for 20 h in a SW41Ti rotor (Beckman Instruments, Fullerton, CA, USA). Following centrifugation, 1 ml fractions were collected from the top of the gradient, yielding a total of 12 fractions. Gradient fractions were resolved by SDS–PAGE on 8% gels and western blot analysis.

### Immunoblot Analysis

Monocytes and macrophages were lysed in RIPA lysis buffer (150 mM NaCl, 10 mM Na_2_HPO_4_, pH 7.2, 1% Nonidet P-40, and 0.5% deoxycholate) containing PMSF (phenylmethylsulfonyl fluoride) (MilliporeSigma), EDTA, and protease and phosphatase inhibitor cocktail (Thermo Fisher Scientific, Waltham, MA, USA). Proteins from supernatants were precipitated using methanol/chloroform. Cell lysates were separated on 8-12% SDS-PAGE gel and transferred onto a PVDF membrane (Bio-Rad, Hercules, CA, USA). The membrane was incubated overnight with the respective primary antibodies at 4°C, and then incubated with peroxidase-conjugated secondary Abs (Cell Signaling Technology) for 1 h at room temperature. The membranes were developed by ECL system. For antibody blocking, anti-hZip6 Ab was pre-incubated with a 2-fold high concentration of blocking peptide (obtained from GW Viteck) in 1 ml of TBS at 4°C for 2 h.

### Immunoprecipitation (IP)

Cell lysates were prepared using modified RIPA buffer (50 mM Tris-HCl, pH 7.4, 150 mM NaCl, 1% Nonidet P-40, and 0.25% deoxycholate) containing PMSF, EDTA, and protease and phosphatase inhibitor cocktail. Dynabead protein A (Thermo Fisher Scientific) were incubated with anti-hZip6 Ab for 1 hr at room temperature and the Ab-conjugated beads were incubated with 1 mg protein lysate at 4°C overnight, followed by washing and elution. The IP products were resolved by SDS-PAGE and immunoblot analysis.

### RT-PCR

Total RNA was extracted from freshly isolated or cultured cells using TRIzol reagents (Life Technologies, Grand Island, NY, USA), and cDNA was synthesized by GoScript reverse transcription system (Promega, Madison, WI, USA). Real-time quantitative RT-PCR was performed in duplicate on a 7500 PCR system (Applied Biosystems, Grand Island, NY, USA). The levels of gene expression were normalized to the expression of β-actin. The comparative CT method (*ΔΔ*CT) was used for quantification of gene expression.

### Intracellular Zinc Measurements With Fluorescent Probes

Cells were incubated in loading buffer [HBBS (-), 1 mM Ca^2+^, 1 mM Mg^2+^, 0.5% BSA] for 30 min, either with 1 μM FluoZin-3-AM (Thermo Fisher Scientific) at 37°C. After washing with washing buffer [PBS + 10% Bovine Serum + 1% Penicillin/Streptomycin] fluorescence was measured by flow cytometry using a BD LSR Fortessa (BD Bioscience) and analyzed using FlowJo software (Tree Star, Ashland, OR).

### Immunological Synapse Formation and Immune Fluorescence Staining

Freshly purified CD4^+^ T cells from healthy donors were pelleted with or without anti-CD3/28 Abs-coated microbeads (Dynabead Human T-Activator CD3/CD28, Thermo Fisher Scientific) by centrifugation, followed by incubation for 30 min at 37°C. Conjugated cells were placed into Menzel Glaser Polysine Adhesion Slides (Thermo Fisher Scientific) for 10 min at 37°C. After washing 3 times with cold PBS, cells were fixed with 4% formaldehyde for 15 min, permeabilized with 0.1% Triton X-100 for 10 min, blocked with 5% normal goat serum in 1 × PBS, and stained with anti-Zip6 Ab (Novus Biologicals) and Zenon Alexa Fluor 568 Mouse IgG (Invitrogen)-labeled anti-CD3 Ab (BD Biosciences) overnight at 4°C. After washing with PBS, cells were incubated with Alexa-488-conjugated goat anti-rabbit IgG (Invitrogen), followed by staining with DAPI (4′,6-diamidino-2-phenylindole). Raji cells were pre-incubated for 30 min with 5 μg/ml of SEE. After washing, Jurkat and SEE-loaded Raji cells were pelleted by centrifugation and incubated for 30 min at 37°C. In some experiments, monocyte-derived mature dendritic cells (mDCs) were pre-incubated with 500 ng/ml of SEB and TSST-1. CD4^+^ T cells were pelleted with SEB/TSST-1-loaded mDCs by centrifugation and incubated for 30 min at 37°C. Subsequent staining was conducted as described above. All images were collected using an Olympus FV3000 confocal microscope (Olympus Corporation, Japan) using a 100 × oil immersion lens.

### Flow Cytometry

Cultured CD4^+^ T cells were stained for 30 min at 4°C with following fluorochrome-conjugated Abs: FITC-anti-CD4 (eBioscience, Santa Clara, CA, USA), PE-anti-CD69, and APC-anti-CD25 (both from BD Bioscience) and 7-AAD (BD Bioscience). The stained cells were acquired using a BD LSR Fortessa (BD Bioscience) and analyzed using FlowJo software (Tree Star, Ashland, OR, USA).

### Statistics

A paired t-test, unpaired t-test, or Pearson correlation analysis was done to analyze data using Prism 7 software (GraphPad Software Inc., La Jolla, CA, USA) as indicated in the figure legends. *P*-Values of less than 0.05 were considered statistically significant.

## Results

### Zip6, a Zinc-Specific Importer, Is Constitutively Localized to Lipid Rafts of Human T Cells

**Figure 1:**
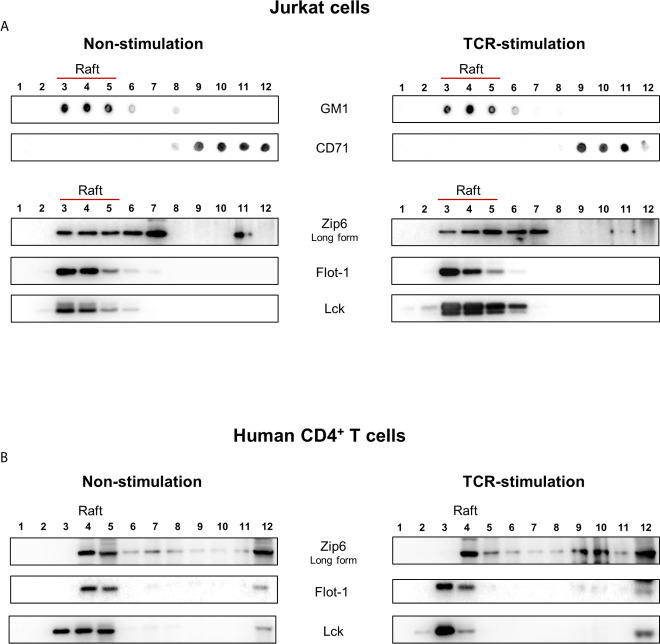
Zip6 is predominantly localized in lipid rafts in human CD4^+^ T cells. Jurkat cells **(A)** and human primary CD4^+^ T cells **(B)** were unstimulated (Left) or stimulated with anti-CD3/CD28 monoclonal antibodies (mAbs) for 15 min at 37°C (Right). Cells were lysed in 1% Triton X-100 buffer and subjected to sucrose density gradients for isolation of the lipid raft fractions. **(A)** Twelve aliquots of fractions collected from the top of the density gradients of unstimulated (Left) or stimulated (Right) Jurkat cell lysates were analyzed by dot blot analysis for GM1, a marker of lipid rafts, and CD71, a marker of non-lipid raft fractions, to identify detergent-resistant membrane fractions containing lipid rafts (Fractions 3-6). Twelve aliquots were further separated by SDS-PAGE and immunoblotted for Lck and Flot-1, additional markers of lipid rafts, and Zip6. **(B)** Twelve aliquots of fractions collected from unstimulated (Left) or stimulated (Right) human primary CD4^+^ T cell lysates were examined by immunoblotting forZip6, Flot-1, and Lck. Data is a representative of three independent experiments with cell lines at different passages or three different healthy donors.

Cytoplasmic zinc concentrations increase immediately after T cell receptor (TCR) triggering, in particular in the subsynaptic compartment. Moreover, this activation-induced zinc influx from extracellular sources depends on the Zip6 transporter in human T cells (16). In agreement with previous studies (16, 17), Zip6 was found to be one of the abundantly expressed Zip molecules in *ex vivo* quiescent CD4^+^ T cells (**Supplementary Figure 1**). Since assembly of the immunological synapse is dependent on the clustering of lipid rafts and is required for optimal T-cell activation, we first examined whether Zip6 resides in lipid rafts of CD4^+^ T cells. Lipid raft composition in Jurkat cells, a human CD4^+^ T-cell line, was examined by lysis with 1% Triton X-100 solution and sucrose gradient ultracentrifugation (**Figure 1A**). Detergent-resistant membrane fractions containing lipid rafts and detergent-soluble fractions (nonlipid rafts) were identified by dot blot analysis and staining with GM1 and CD71, respectively, showing an efficient segregation of the two fractions (**Figure 1A**). To further analyze lipid raft fractions, we carried out SDS-PAGE followed by immunoblot analysis to detect flotilin-1 and Lck, molecules known to be resident in T cell lipid rafts. Lipid raft markers, including GM, Flotilin-1 and Lck, were present in fractions 3-6 from Jurkat cells, whereas CD71 was detected in fractions 9-12 (**Figure 1A**). To test whether Zip6 is localized in lipid rafts, the same twelve aliquots of fractions from Jurkat cell lysates were examined by immunoblotting using anti-Zip6 antibody. Our data illustrate that Zip6 is mainly localized in the detergent-resistant membrane (DRM) lipid raft fractions (**Figure 1A** and **Supplementary Figure 2**). As shown in **Figure 1B**, this finding was confirmed in freshly purified human CD4^+^ T cells. It has been known that Zip6 exists as two forms, one 755 amino acids (aa) long and a 433 aa short-form (21). Zip6 residing in lipid rafts of primary CD4^+^ T cells mainly belonged to long-form. Some Zip6 was also present in faction 12 where Lck exists, showing a substantial overlap between Zip6 and Lck in Jurkat cells and primary CD4^+^ T cells regardless of TCR triggering (**Figure 1**). These findings demonstrate that Zip6 constitutively localizes in lipid rafts of human CD4^+^ T cells.

### Zip6 Is Accumulated Into the Immunological Synapse in CD4^+^ T Cells Upon TCR Stimulation

**Figure 2:**
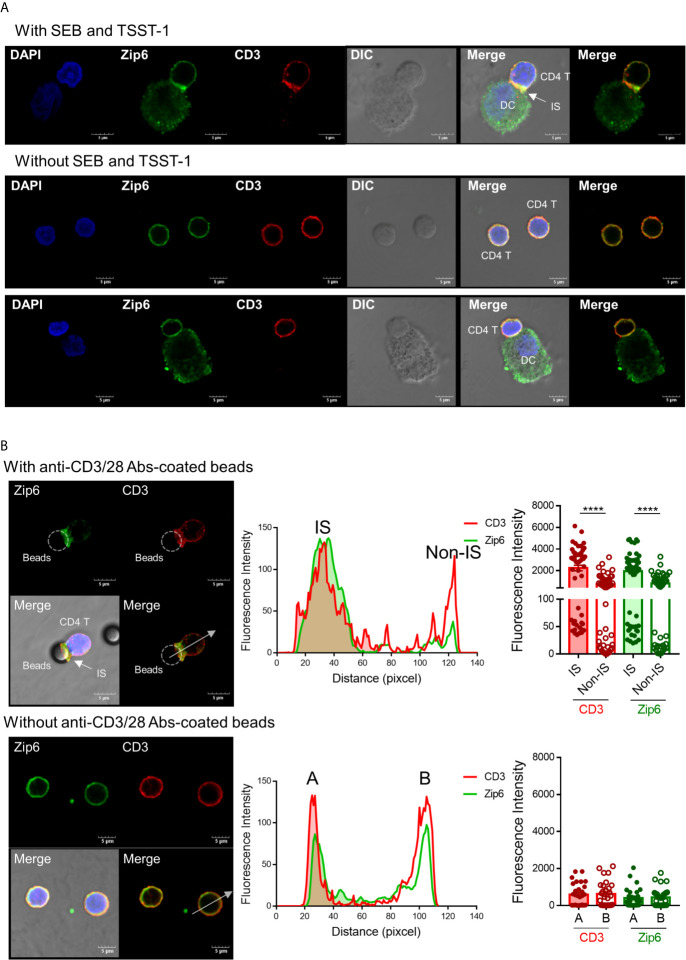
Zip6 is recruited into the immunological synapse of stimulated T cells. **(A)** Monocyte-derived DCs were loaded with or without SEB and TSST-1 (500 ng/ml) for 30 min and pelleted with primary CD4^+^ T cells by centrifugation followed by incubation for 30 min at 37°C. Cells were allowed to adhere to poly-lysine coated glass slides, and fixed, permeabilized and stained for CD3 (red) and Zip6 (green), and analyzed by confocal microscopy. Arrow indicates the accumulation of Zip6 and CD3 at the established immunological synaptic area. **(B)** Human CD4^+^ T cells were pelleted with or without anti-CD3/28 antibody-coated microbeads by centrifugation, followed by incubation for 30 min at 37°C. Cells were fixed, permeabilized, and stained with anti-Zip6 (green) and CD3 (red) antibodies, and analyzed by confocal microscopy. Accumulation of Zip6 (green) and CD3 (red) was quantified by fluorescence along a cross section perpendicular to the synapse (white dotted line arrow in left panel) as line-intensity histograms (middle panel). Fluorescence intensity of CD3 and Zip6 at the site of T cell-microbeads interaction was dramatically increased in human CD4^+^ T cells (right panel). Results are from three independent experiments. Scale bars represent 5 μm. Bar graphs show the mean ± S.E.M. **** = *p* < 0.0001 by two-tailed paired *t*-test.

TCR signaling molecules are recruited to lipid rafts at the junction between T cells and APCs, an area known as the immunological synapse (22, 23). Given that Zip6-mediated influx of zinc is mostly confined to the subsynaptic area in T cells, we sought to test whether Zip6 is accumulated into the immunological synapse of CD4^+^ T cells upon TCR stimulation. To this end, Jurkat cells were conjugated with Raji cells, a human B lymphoblastoid cell line, preincubated with super-antigen Staphylococcus Enterotoxin E (SEE) and localization of Zip6 was analyzed using confocal microscopy. We foundthat Zip6 accumulates at the contact area between Jurkat and SEE loaded-Raji cells where CD3, used as a marker for the immunological synapse, is also assembled (**Supplementary Figure 3A** upper panel). Jurkat cells not in contact with Raji cells had Zip6 and CD3 evenly distributed over their entire membrane similarly to Jurkat cells cocultured with Raji cells treated without SEE (**Supplementary Figure 3A** lower panel). Consistent with the findings in Jurkat cells, Zip6 was predominantly accumulated into the immunological synapse assembled in the contact area between human primary CD4^+^ T cells and mature monocyte-derived dendritic cells (DC) pre-loaded with the super-antigen toxic shock syndrome toxin (TSST) and staphylococcal enterotoxin B (SEB) (**Figure 2A**). Since Zip6 is also expressed in Raji cells and mature DCs, it cannot be definitively concluded that the Zip6 present in the IS is derived from Jurkat or CD4^+^ T cells. To overcome this technical difficulty, human CD4^+^ T cells were activated with anti-CD3/28 coated Dynabeads and the accumulation of Zip6 into immunological synapse was visualized (**Figure 2B**). As shown in the tracing of Zip6 and CD3 fluorescence along the cross section perpendicular to the synapse plane, there was increased Zip6 colocalization with the CD3 cluster at the immunological synapse (**Figure 2B** upper panel), while expression of Zip6 and CD3 was evenly distributed in unstimulated CD4^+^ T cells (**Figures 2A**, B lower panel). Our findings were corroborated by the colocalization of Zip6 and GM1 at immunological synapse in CD4^+^ T cells upon TCR stimulation (**Supplementary Figure 3B**). These data suggest that Zip6 is accumulated along with lipid rafts into the immunological synapse in CD4^+^ T cells upon TCR stimulation.

### Zip6 Is Phosphorylated by Zap70 in Response to TCR Stimulation

Considering that Zip6-mediated zinc influx in T cells depends on TCR stimulation, the finding of Zip6 localization to the IS of activated T cells prompted us to investigate whether Zip6 activity is regulated by TCR signal transduction. *In silico* phosphosite prediction revealed one single and two adjacent tyrosine residues of Zip6 with potential for phosphorylation [Y493, Y528 and Y531 (https://www.phosphosite.org/homeAction)]. The presence of these residues in the long cytoplasmic loop of Zip6 suggests they may be involved in its activation. Therefore, to explore the mechanism by which Zip6 activation is regulated, we first sought to investigate whether the phosphorylation of Zip6 was regulated by TCR stimulation. Since no commercially available anti-Zip6 antibodies (Abs) are currently available for immunoprecipitation of Zip6, we developed a polyclonal antibody applicable to immunoprecipitation assay. This in-house Zip6 polyclonal Ab was developed to recognize a consensus sequence (QNHHPHSHSQRYSREELKDAG: 571-591 and 296-316 of full- and short-form, respectively) present in both Zip6 isoforms (**Supplementary Figure 4A**). As demonstrated in **Supplementary Figures 4B, C**, our in-house Zip6 Ab detected two major target bands at around 98 and 60 kDa in Jurkat cell lysates and clearly recognized isoforms at 95 and 70 kDa in the lysates of primary human CD4^+^ T cells. The specificity of our polyclonal Zip6 Ab was evaluated by pre-incubating with a blocking peptide comprised of the sequence used to generate the antibody (**Supplementary Figures 4B, C**). Finally, we confirmed that our human Zip6 antibody was applicable to IP experiments (**Supplementary Figure 4D**), showing that two target bands of around 98 and 60 kDa, corresponding to Zip6 isoforms, were identified in the Zip6 pulldown lane and input lane, but not in the normal rabbit IgG control lane on immunoblot. These data demonstrate that our in-house Zip6 Ab can efficiently and specifically immunoprecipitate Zip6 from cell extracts (**Supplementary Figure 4D**)

**Figure 3:**
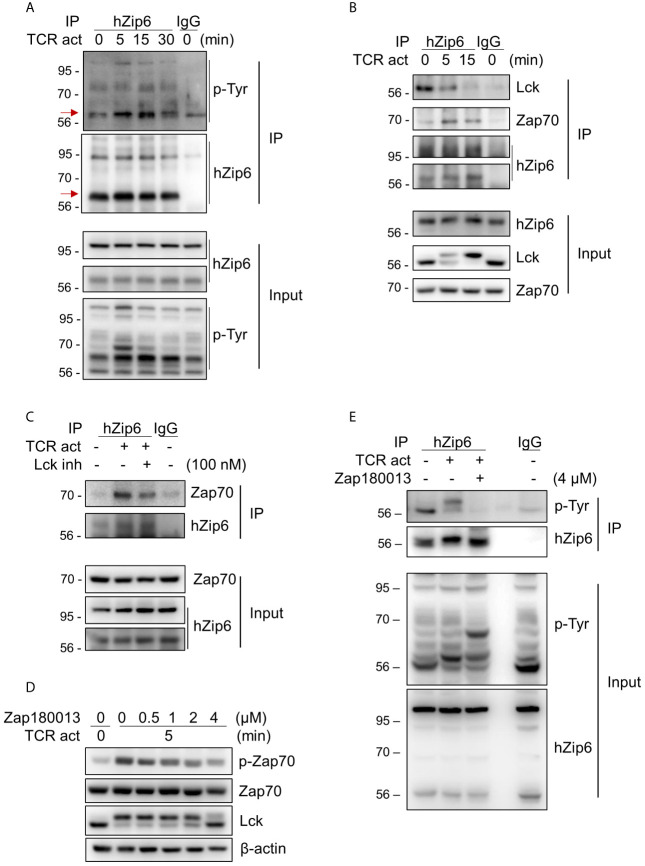
Zip6 is phosphorylated by Zap70 in response to TCR stimulation. Jurkat cells were coated with anti-CD3 and anti-CD28 Abs, followed by cross-linking with goat anti-mouse IgG for the indicated times and under the indicated conditions at 37°C. The lysates were prepared and immunoprecipitated with our in-house anti-human Zip6 Ab (hZip6) or control IgG. **(A)** Immunoprecipitates (IP) were analyzed by immunoblot analysis to detect tyrosine phosphorylation of Zip6. Data are representative of experiments replicated at least three times. Red arrows indicate target bands **(B)** Jurkat cells were stimulated, lysed, and immunoprecipitated with hZip6 Ab or control IgG as described in **(A)**. Immunoprecipitates were analyzed by immunoblot analysis to detect Lck and Zap70. Data are representative of three experiments. **(C)** Jurkat cells were stimulated by CD3/CD28 cross-linking for 30 min in the presence of Lck inhibitor (RK-24466; 100 nM) to block phosphorylation of Zap70, lysed and immunoprecipitated with hZip6 Ab or control IgG as described in **(A)**. Immunoprecipitates were analyzed by immunoblot analysis to detect Zap70. Data are representative of three experiments. **(D)** Jurkat cells were pre-treated for 30 min with the indicated concentration of Zap70 inhibitor (Zap180013), followed by stimulation with CD3/CD28 cross-linking for 5 min. Cell lysate were prepared for immunoblotting. **(E)** Jurkat cells pretreated with or without 4 μM of Zap180013 were stimulated with CD3/CD28 cross-linking for 15 min, lysed, and immunoprecipitated with hZip6 Ab or control IgG. Immunoprecipitates were analyzed by immunoblot analysis to detect phospho-tyrosine.

To investigate whether Zip6 is phosphorylated in T cells following TCR activation, we performed immunoprecipitation assays using our in-house Zip6 Ab on lysates from Jurkat cells stimulated with anti-CD3 and anti-CD28 Abs followed by cross-linking with a secondary Ab. As seen in **Figure 3A**, immunoprecipitation followed by immunoblotting using pan-phosphotyrosine Ab clearly showed increased phosphorylation of Zip6 tyrosine residues from 5 minutes after TCR triggering, an effect that diminished 30 min after TCR triggering (**Figure 3A**). Considering that Zip6-mediated zinc influx and phosphorylation of Zip6 occurred very early after TCR stimulation (**Figure 3A**) (16), we hypothesized that Zip6 is phosphorylated by kinases that participate in proximal TCR signaling. *In silico* analysis of kinase-specific phosphorylation sites in proteins using GPS 5.0 and KinasePhos predicted Y493, Y528, and Y531 residues to be phosphorylated by Src and/or Syk kinases. Since Lck and Zap70 are the main Src- and Syk family kinases, respectively, involved in proximal TCR signaling, proteins immunoprecipitating with anti-Zip6 Ab were analyzed by immunoblotting using anti-Lck and anti-Zap70 Abs (**Figure 3B**). TCR activation markedly enhanced the interaction between Zap70 and Zip6 in Jurkat cells in a time-dependent manner until 15 min post-cross-linking, while the Zip6-Lck association was rather reduced following TCR stimulation (**Figure 3B**). This finding was also confirmed using primary human CD4^+^ T cells (**Supplementary Figure 5**). To further investigate whether the interaction between Zip6 and Zap70 is dependent on TCR signal transduction, an Lck-specific inhibitor (RK-24466) and Zap70 inhibitor (Zap 180013) were utilized (24–26). At concentrations as low as 10 nM this Lck inhibitor efficiently abated phosphorylation of Zap70 (**Supplementary Figures 6A, B**). Further, as seen in **Figure 3C**, RK-24466 also suppressed the interaction between Zip6 and Zap70 in Jurkat cells upon TCR stimulation. Zap 180013 repressed the phosphorylation of Zap70 in TCR-stimulated Jurkat cells with dose-dependent manner (**Figure 3D**) and tyrosine phosphorylation of Zip6 was inhibited by treatment with Zap180013 (**Figure 3E**). These data suggest that activation of Zip6 is closely associated with proximal TCR signaling and is regulated by Zap70-mediated phosphorylation during TCR stimulation.

### Zip6-Mediated Zinc Influx Is Induced by TCR Activation in CD4^+^ T Cells

**Figure 4:**
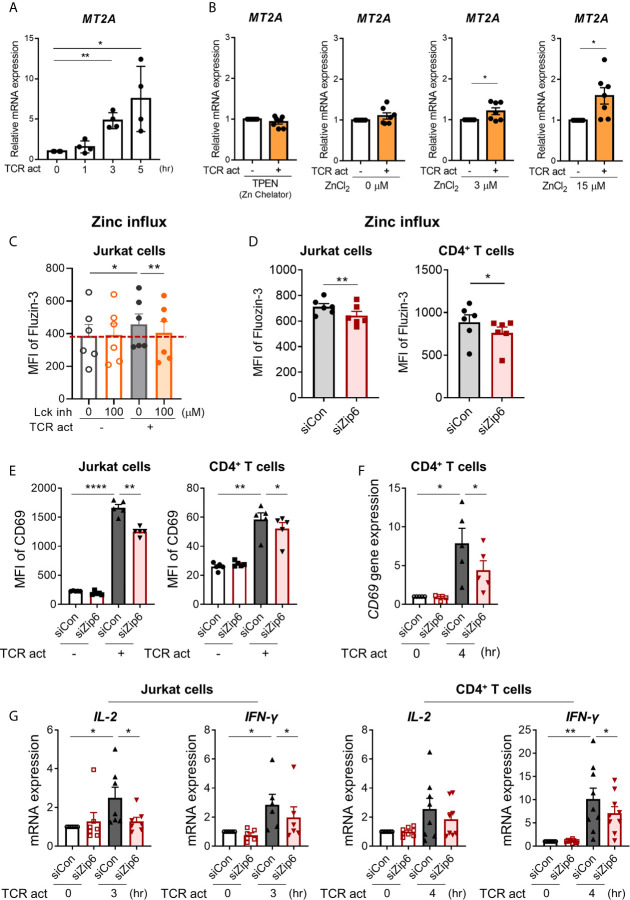
Zip6-mediated zinc influx is induced by TCR activation in CD4^+^ T cells. Freshly purified human CD4^+^ T cells were coated with anti-CD3 and anti-CD28 Abs, followed by cross-linking with goat anti-mouse IgG for the indicated times and under the indicated conditions at 37°C **(A)** Metallothionein 2A (MT2A) mRNA was quantified by real-time RT-PCR at the indicated time points (*n* = 4). **(B)** Human CD4^+^ T cells were stimulated by crosslinking for 3 h in the presence of 1.5 μM TPEN (TP) or different concentrations of ZnCl_2_ (0, 3, and 15 μM). MT2A mRNA level was quantified by real-time RT-PCR (*n* = 7). **(C)** Jurkat cells were pretreated with RK-24466, a Lck inhibitor for 30 min at 37°C, followed by stimulation by CD3/CD28 cross-linking for 30 min at 37°C. Cells were stained with FluoZin-3, a zinc-specific fluorescent probe and its fluorescent intensity was analyzed by flow cytometry. MFI is a value between stained cells versus non-stained cells. **(D)** Jurkat cells (left) and human CD4^+^ T cells (right) were transfected with scrambled control siRNA (siCon; black line bar) or Zip6 siRNA (siZip6; red line bar) for 48 h. Cells were pre-loaded with FluoZin-3, a zinc-specific fluorescent probe, and coated for 30 min with anti-CD3 and anti-CD28 Abs, followed by cross-linking with anti-mouse IgG for 30 min at 37°C (n = 6). Fluorescent intensity of FluoZin-3 was analyzed by flow cytometry. **(E–G)** Jurkat cells and CD4^+^ T cells transfected with control (siCon; black line bar) or Zip6 siRNA (siZip6; red line bar) were stimulated with anti-CD3/CD28 for 24 h **(E)** or 3h **(F, G)**, followed by analysis of CD69 expression using flow cytometry or analysis of CD69, IL-2 and IFN-γ mRNA expression by real-time RT-PCR, respectively. Expression was normalized to β-actin, and the comparative Ct method was used for quantification of gene expression **(A, B, F, G)**. Bar graphs show the mean ± S.E.M. *p < 0.05, **p < 0.01, and ****p < 0.0001 by two-tailed paired *t*-test.

To examine whether increased cytoplasmic zinc is attributable to Zip6-mediated zinc influx from extracellular sources, we monitored the expression of metallothionein 2A (MT2A), a major zinc-binding protein that plays coordinated roles in the distribution, transport, and maintenance of intracellular zinc (27, 28). Furthermore, the induction of metallothionein (MT) expression is largely dependent on the increase of intracellular zinc (29). Reflecting the rapid influx of zinc (16), expression of MT2A mRNA began to increase within 3 h after TCR stimulation (**Figure 4A**) in a manner largely dependent on extracellular zinc concentration (**Figure 4B**). Further, MT2A expression was minimal in zinc-depleted medium or without TCR stimulation. This finding was corroborated by blockage of early TCR signaling with the Lck inhibitor. RK-24466, a Lck-specific inhibitor, suppressed zinc influx and the induction of MT2A in TCR-activated CD4^+^ T cells at 0.5 and 3 h post-stimulation, respectively, indicating reduced zinc influx by inhibition of early TCR signaling (**Figure 4C** and **Supplementary Figure 6C**). Moreover, Zap70 inhibitor completely suppressed the induction of MT2A in TCR-stimulated CD4^+^ T cells (**Supplementary Figure 6D**). To further examine the role of Zip6 on zinc influx and T cell responses, Jurkat cells were transfected with Zip6-targeted or scrambled siRNA and stimulated with anti-CD3 and anti-CD28 Abs. As depicted in **Supplementary Figure 7**, by qPCR and immunoblotting, Zip6 of Jurkat and human CD4^+^ T cells was efficiently silenced (80~90%) and this led to a significant reduction of intracellular zinc as measured by FluoZin-3, a zinc specific-fluorescent probe (**Figure 4D**). Lastly, we sought to explore the effect of Zip6-mediated zinc influx on T-cell activation. After transfection with Zip6-targeted siRNA (**Supplementary Figure 7**), T cell responses, such as activation markers and cytokine induction, were analyzed in Jurkat cells and primary CD4^+^ T cells after TCR triggering. Zip6 silencing led to a repression of CD69 expression in TCR-activated Jurkat cells and primary CD4^+^ T cells (**Figures 4E, F**). Moreover, IL-2 and IFN-γ gene expression were also diminished at an early time-point due to reduced zinc influx caused by Zip6 silencing as described previously (**Figure 4G**) (7). These data demonstrate that TCR-mediated Zip6 activation contributes to zinc influx, which is important for T-cell activation.

## Discussion

Zinc is a fundamental micronutrient and the second most plentiful trace metal in the human body. Recently the multifaceted role of zinc in the regulation of innate and adaptive immune responses has become appreciated. Zinc deficiency, mainly caused by malnutrition, is linked with cell-mediated immune dysfunction. Besides its classical role as a cofactor for enzymes and transcription factors, identification of several molecular targets, including phosphatases, phosphodiesterases and kinases, suggests that similar to calcium, zinc is also a second messenger/signaling ion that regulates signal transduction in various immune cells (30, 31). Zinc homeostasis is tightly controlled by families of 14 Zips and 10 ZnTs that are spatiotemporally expressed in the plasma membrane or in the membranes of intracellular organelles (32). The tissue- and cell-specific expression of the various Zips and ZnTs allows the involvement of specific transporters in the regulation of cellular responses including immune responses. Zip6 and Zip8 have been established as major transporters playing immunoregulatory roles in T-cell responses (7, 16, 17). Recent studies have suggested that TCR activation-induced zinc influx is mediated by the zinc transporter Zip6, which is primarily expressed in the plasma membrane of T cells, and increased zinc influx augments TCR signaling. Of note, the Zip6-mediated increase in cytoplasmic zinc occurs within minutes after TCR stimulation and this increase is not diffuse, but rather, is compartmentalized in the subsynaptic area. However, the mechanism underlying these finding remains poorly understood. Thus, we hypothesized that Zip6 participates in formation of immunological synapse (IS) upon TCR stimulation and its activation is associated with proximal TCR signaling.

Cytoplasmic zinc exerts immunomodulatory roles *via* direct and/or indirect influences on TCR signaling molecules. Early studies suggested that the binding of Lck to CD4 or CD8α is dependent on zinc, indicating a structural role for this ion in the TCR signaling complex (33, 34). The src-family kinase Lck, an essential kinase in early TCR signaling events, is recruited to the TCR upon stimulation and associates with CD4 or CD8α then phosphorylates the TCR ζ chain, an initiation step in TCR signaling. Accumulating evidence demonstrates that zinc ions inactivate tyrosine phosphatases such as protein tyrosine phosphatases (PTPs) 1b (35) and PP2A (36) and serine/threonine phosphatases including calcineurin (7) by yet unidentified mechanisms (37). Furthermore, it has recently been shown that recruitment of SHP-1, a PTP, to the TCR synapse is reduced with increasing zinc influx upon TCR stimulation, probably due to zinc-induced steric conformational changes in SHP-1 that prevent binding to Lck. SHP-1 is important for dephosphorylation of tyrosine motifs of early TCR signaling molecules (38). Thus, the zinc-mediated inhibition of phosphatases conserves phosphorylation of signaling molecules and generally sustains signaling activity (7, 16, 35). Considering its immunoregulatory effects on a variety of signaling pathways in T cells, zinc homeostasis is tightly controlled by transporters such as Zip6 and Zip8, and MT, a zinc-binding/buffering protein, during T cell responses.

In agreement with previous studies (16, 17), our study underscores that Zip6 is a pivotal component of the T cell activation machinery. Among 14 Zip molecules, Zip6, 8, and 13 are plentifully expressed in human CD4^+^ T cells (**Supplementary Figure 1**). Zip6 is known to be mainly located in the plasma membrane of cells, whereas Zip8 and Zip13 are predominantly expressed on the lysosome and ER/Golgi membrane, respectively (7, 16, 39). Considering that activation-induced zinc homeostasis in human T cells is influenced by extracellular zinc concentrations (**Figure 4B**) (7, 16), Zip6 was identified as a potential candidate in the present study. The monitoring of intracellular zinc ions using FluoZin-3 illustrated that in Zip6 knockdown (KD) Jurkat cells and CD4^+^ T cells, zinc influx was significantly reduced upon TCR stimulation (**Figure 4D**), suggesting a role for Zip6 as a major zinc importer in TCR-activated T cells. Functionally, Zip6 silencing leads to repression of T cell activation as determined by CD69 expression (**Figures 4E, F**), presumably *via* inhibition of Zip6-mediated zinc influx. However, zinc influx and associated reduction of CD69 expression were less noticeable compared to a previous finding using a T cell-DC-superantigen system (16). In the present study, T cells were activated by CD3/CD28 cross-linking for biochemical analysis of the TCR signaling components-Zip6 complex. This again emphasizes the importance of physiological IS formation for the immunoregulatory role of Zip6. Our results also confirmed impaired expression of IL-2 and IFN-γ in Zip6 KD Jurkat cells and IFN-γ in Zip6 KD CD4^+^ T cells (**Figure 4G**), indicating the importance of Zip6-mediated zinc influx for TCR activation (17). Zip8, a major Zip protein in T cells, transports zinc from the lysosome to cytoplasm during T-cell activation and Zip8-mediated increase of cytoplasmic zinc results in increased production of IFN-γ (7). These findings suggest that the expression and function of Zip proteins contribute to the TCR-mediated T cell activation process.

MT synthesis is predominantly controlled by intracellular zinc, which enables metal regulatory transcription factor 1(MTF-1) to bind to the multiple metal response elements in the MT promoter (27). It has been shown that external zinc concentrations cause increased MT in activated T cells, but not in non-activated T cells even in the same culture. Moreover, this increase is dependent on Zip6-mediated zinc influx (29). Our data clearly shows that the expression of MT2A, a member of the MT family, is upregulated as early as 3 hr after TCR stimulation in a zinc concentration-dependent manner (**Figures 4A, B**). We confirmed that MT2A expression was minimally changed by TCR stimulation in zinc-depleted culture medium, confirming that TCR activation-induced zinc influx from external sources is important for T-cell activation. Of note, blockage of early TCR signaling events using an Lck inhibitor or Zap70 inhibitor completely repressed MT2A expression (**Supplementary Figures 6C, D**), suggesting that Zip6 activation is largely dependent on TCR stimulation. However, the molecular mechanisms regulating the induction of Zip6-mediated zinc influx during early TCR signaling are not yet known.

Lipid rafts, which are cholesterol and glycosphingolipid-enriched microdomains of plasma membranes, serve as important platforms for signal transduction (40). A large number of studies have suggested that an intimate link between lipid rafts and immunoreceptor (such as TCR) signaling is critical for T cell activation (41). Several signaling proteins that participate in the TCR signaling network, such as Lck, LAT, CD3ζ and CD4, are highly enriched in lipid rafts of resting T cells (42, 43). Upon TCR triggering lipid rafts reorganize and signaling proteins in the rafts are recruited into the site of T cell engagement with antigen presenting cells, the immunological synapse (IS). Here TCR signal transduction is facilitated by the increased concentration of many receptors and signaling molecules at the center of the T cell-APC contact (22, 44). An intriguing finding in the present study is that that Zip6 is predominantly localized within lipid rafts of human CD4^+^ T cells (**Figure 1**). Further, TCR stimulation leads to accumulation of Zip6 in the IS (**Figure 2**). Thus, our findings may give an explanation as to why the Zip6-mediated increase in cytoplasmic zinc is not diffuse, but is instead compartmentalized in the subsynaptic area, as depicted in a previous report (16).

Similar to Zip6, TCR-induced redistribution of several ion channels into the IS has been reported. For example, Kv1.3 potassium channels, which are essential participants in T-cell activation, are localized to the IS (45). Orai1 and STIM1 (46), which are the calcium release-activated calcium (CRAC) channel pore subunit and its activator, respectively, and P2X1 and P2X4 receptors (47), which act as ATP-gated Ca^2+^ channels, are also rapidly recruited into the IS, where they contribute to Ca^2+^ influx as an ionic signaling molecule in activated T cells (48). Given the important roles of various metal ions, including Ca^2+^, Zn^2+^ and K^+^, for regulating immune responses, especially *via* the regulation of signaling pathways, it is clear ion channels and transporters are important elements for efficient transmembrane TCR signaling in the IS.

The Zip family consists of thousands of evolutionarily conserved, integral membrane proteins in plants, animals, and even in prokaryotes. Most Zip proteins are predicted to contain eight transmembrane domains (TMDs) and one variable large cytoplasmic loop between TM3 and TM4. Because the 3-D structure of full-length human Zip proteins remains to be solved, structural information on zinc transporters has been gleaned from sequence analysis and prediction studies as well as the crystal structures of prokaryotic homologs (18, 19, 49, 50). Thus, the exact molecular mechanisms regulating Zip activities and mediating zinc influx by Zip proteins remain controversial.

Taylor and colleagues first proposed that protein phosphorylation is likely to be crucial to the regulation of zinc transporter activity (20). This study revealed that in the breast cancer MCF-7 cell-line, phosphorylation of Zip7, which resides in the ER membrane, is regulated by the protein kinase CK2 (casein kinase 2) and is linked to the release of the free zinc from intracellular stores into the cytoplasm (20). Zip7-mediatated increases in intracellular zinc are involved in activation of Akt and ERK1/2, leading to effects on cell proliferation and migration. Of note, *in silico* sequence analyses predict all 24 human zinc transporters to be phosphorylated, underscoring the role of protein phosphorylation as an important mechanism controlling transporter function (49). Our phosphosite prediction analysis suggested a high probability of phosphorylation of tyrosine residues, Y493, Y528 and Y531, in its cytoplasmic loop between TM3 and TM4 of Zip6. Importantly, tyrosine phosphorylation of Zip6 was found to be increased from 5 min post-TCR stimulation *via* an interaction with Zap70 (**Figure 3**), an essential kinase of early TCR signaling. These findings and others suggest that the activity of Zip proteins can be modulated by phosphorylation as part of cellular signaling processes (20, 49). There are additional putative tyrosine and serine-threonine phosphorylation sites on Zip6. Thus, a site mutation study will be necessary to definitively conclude which residues are phosphorylated and involved in the activation of Zip6 in T cells. We believe the three tyrosine residues found in our analysis are likely the key residues since no obvious phosphorylation of serine was seen at early time points following TCR triggering (data not shown) and phosphorylation of residues within the cytoplasmic loop between TM3 and TM4 are likely to be important for interaction with signaling proteins at the IS. A recent study has shown that the large cytoplasmic loop between TM3 and TM4 is an intrinsically disordered protein region (IDPR). IDPRs are known to play critical biological roles for cell signaling, regulation, and control by serving as a protein-specific regulatory domain, although they lack secondary and tertiary structural elements (51).

Our findings help reveal how local zinc influx occurs after TCR stimulation, and how this serves to specifically amplify early TCR signals without globally modifying the various cellular processes controlled by zinc. To summarize, upon TCR triggering Zap70 activates Zip6, which is localized to the IS, through phosphorylation of tyrosine residues likely located in the long cytoplasmic loop. Activated Zip6 rapidly induces local zinc influx in the subsynaptic region. Cytoplasmic zinc ions inhibit recruitment of SHP-1 to the TCR synapse (16), and thereby may be involved in regulation of Lck activity due to phosphorylation of an inhibitory tyrosine at the C terminus (position Y505) controlled by PTP CD45 and the Csk kinase. Moreover, Csk kinase activity has been shown to be magnesium dependent and inhibited by zinc (52).

In summary, the current study provides new insight into the crosstalk between Zip6 activity and early TCR signaling events during T-cell activation. Here, we investigated the molecular mechanisms underlying TCR-mediated Zip6 activation that induce local zinc influx in the vicinity of the IS of T cells. Zip6, which primarily resides in lipid rafts, participates in formation of the IS upon TCR stimulation, and TCR stimulation leads to tyrosine phosphorylation of Zip6, mediated through its physical interaction with Zap70. The reduction of extracellular zinc influx in Zip6-silenced T cells resulted in decreased T-cell activation as measured by the expression of CD69, IL-2, and IFN-γ. Together, these findings increase understanding of the spatiotemporal mechanisms controlling zinc homeostasis and the resultant effects on T cell activation.

## Data Availability Statement

The original contributions presented in the study are included in the article/**Supplementary Material**. Further inquiries can be directed to the corresponding author.

## Ethics Statement

The studies involving human participants were reviewed and approved by the institutional review board of Seoul National University Hospital. The patients/participants provided their written informed consent to participate in this study.

## Author Contributions

BK participated in the design of the study, performed most of the experiments, data collection and analysis, and drafted the manuscript. HK participated in the design of the study, performed the experiments. W-WL conceived of the study, participated in its design and coordination, performed data analysis and writing of the manuscript, and has full access to all the data in this study and provided financial support. All authors contributed to the article and approved the submitted version.

## Funding

This work was supported by a grant (NRF-2018R1A2B2006310 to W-WL) from the National Research Foundation of Korea (NRF) funded by Ministry of Science and ICT (MSIT), Republic of Korea.

## Conflict of Interest

The authors declare that the research was conducted in the absence of any commercial or financial relationships that could be construed as a potential conflict of interest.

## Publisher’s Note

All claims expressed in this article are solely those of the authors and do not necessarily represent those of their affiliated organizations, or those of the publisher, the editors and the reviewers. Any product that may be evaluated in this article, or claim that may be made by its manufacturer, is not guaranteed or endorsed by the publisher.
