# Supplementary material for: Zap70 Regulates TCR-Mediated Zip6 Activation at the Immunological Synapse
Source: Front Immunol. 2021 Jul 29;12:687367. doi: 10.3389/fimmu.2021.687367 (PMC8358678; doi:10.3389/fimmu.2021.687367)
Supplement: Supplementary file 1 [file DataSheet_1.pdf]

Zap70 regulates TCR-mediated Zip6 activation at the immunological synapse

Bonah Kim, Hee Young Kim, and Won-Woo Lee

Supplementary Figure 1

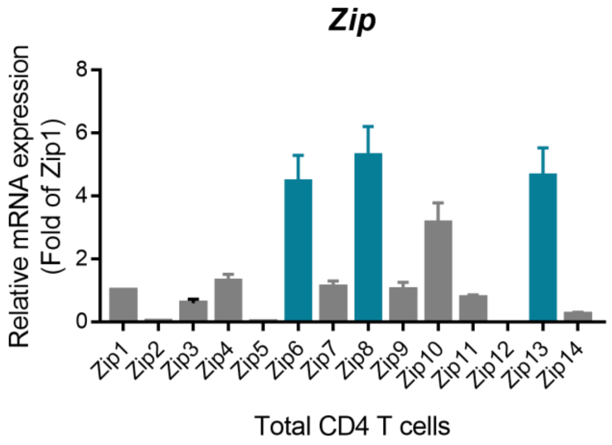

Figure 1. Expression of Zip family members in total CD4 T cells
Expression of 14 ZIP family genes was quantified by real-time RT-PCR in $CD4^{+}$T cells (n=10) freshly isolated from PBMCs. Relative expression ZIP mRNAs was normalized to Zip1.

Supplementary Figure 2

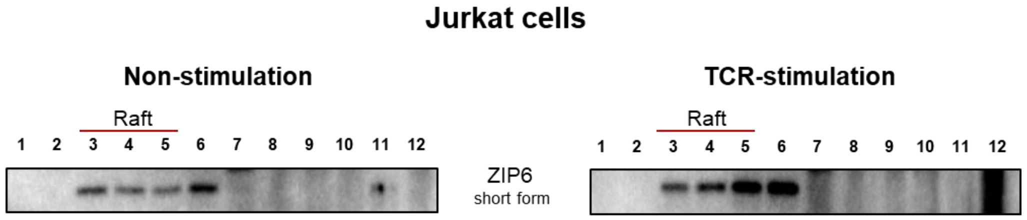

Figure 2. Zip6 is localized in lipid rafts

Jurkat cells were unstimulated (Left) or stimulated with anti-CD3/CD28 monoclonal antibodies (mAbs) for 15 min at 37°C (Right). Cells were lysed in 1% Triton X-100 buffer and subjected to sucrose density gradient for isolation of lipid raft fractions. Twelve aliquots were further separated by SDS-PAGE and immunoblotted for Lck and the short form of Zip6

Supplementary Figure 3

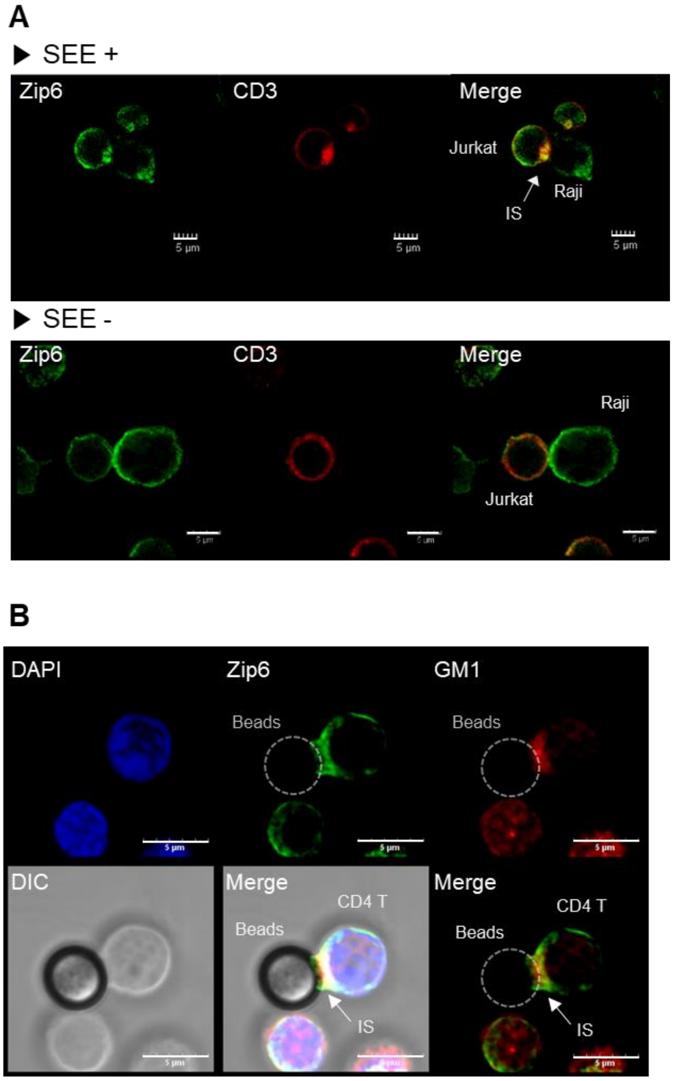

Figure 3. Accumulation of ZIP6 into the immunological synapse of stimulated T cells. **(A)** Raji cells were loaded with or without 1 µg SEE for 30 min and pelleted with Jurkat cells by centrifugation, followed by incubation for 15 min at 37°C. Cells were allowed to adhere to poly-lysine coated glass slides then fixed, permeabilized, and stained for CD3 (red) and Zip6 (green) for analysis by confocal microscopy. Arrow indicates the accumulation of Zip6 and CD3 at established immunological synaptic area. (**B**) Freshly isolated human $CD4^+$ T cells were pelleted with anti-CD3/28 Abs-coated microbeads by centrifugation, followed by incubation for 30 min at 37°C. Cells were fixed, stained with CTX-B-Alexa 555 (red), permeabilized, and then stained with anti-Zip6 (green) Ab. Results are representative of four independent experiments. Scale bars represent 5 µm.

Supplementary Figure 4

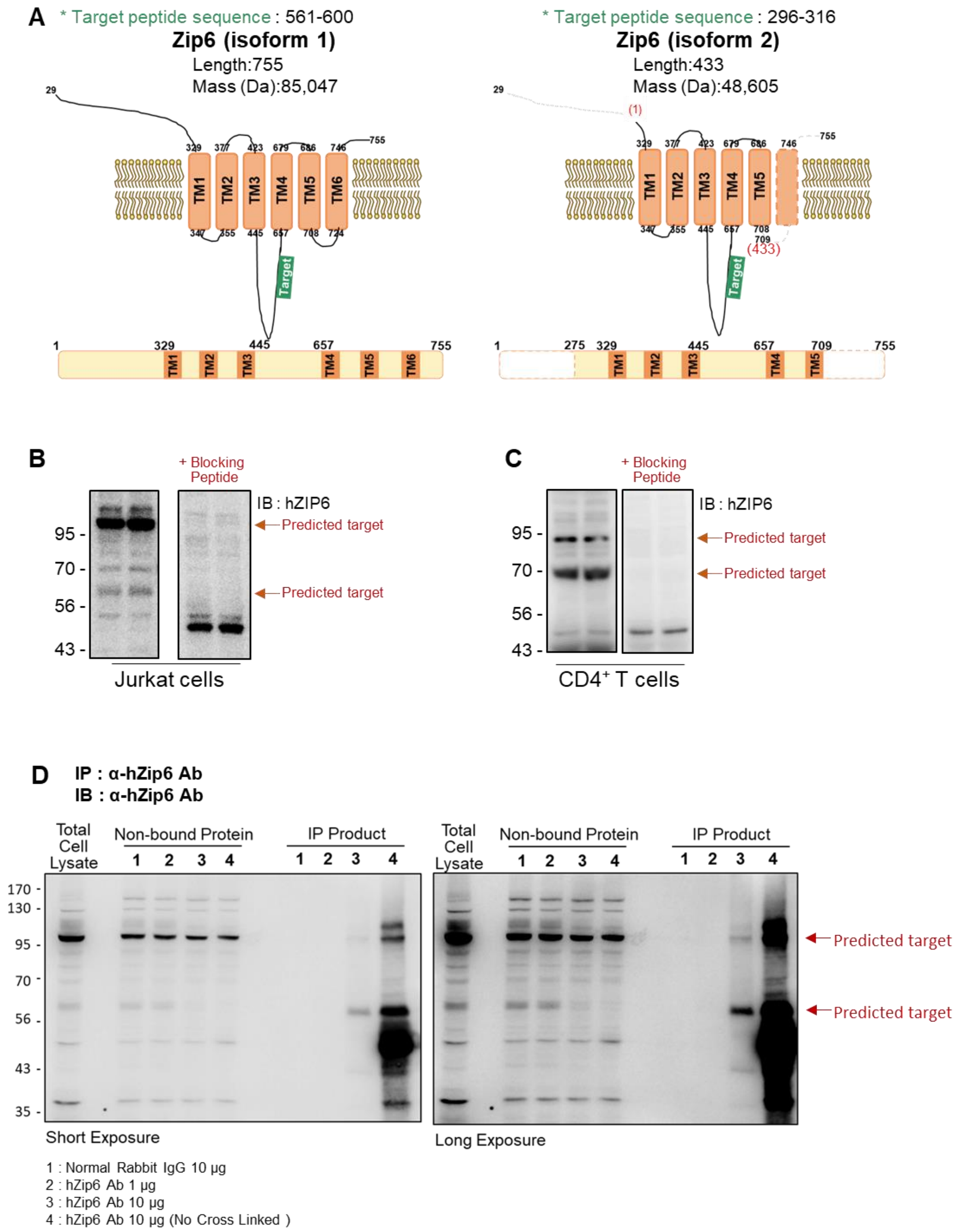

Figure 4. Development of polyclonal anti-human Zip6 antibody

(A) Schematic diagram depicting structure of Zip6 (SLC39A6) and the target peptide. Antibody was raised against amino acid residues 561-600 of isoform 1 (Full form) of Zip6. **(B, C)** Jurkat (B) and primary $CD4^+$ T (C) cell lysates were immunoblotted with the human Zip6 antibody preincubated at 1:2 ratio at 4°C for 4 hr with blocking peptide. **(D)** Jurkat cell lysates were immunoprecipitated (IP) with human Zip6 antibodies and immunoblotted with the same human Zip6 antibody to confirm applicability for immunoprecipitation assays.

Supplementary Figure 5

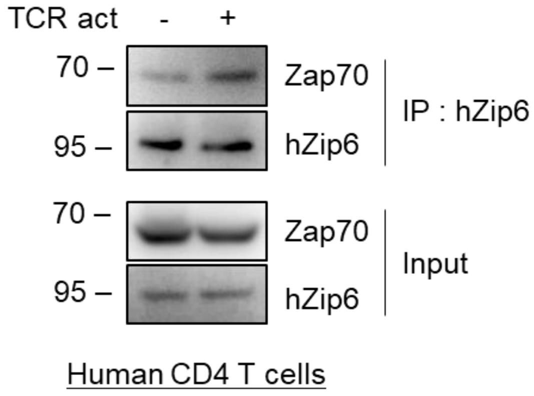

Figure 5. Increased association of Zap70 with Zip6 by TCR stimulation in primary human CD4$^+$ T cells.

Purified CD4$^+$ T cells were coated with anti-CD3 and anti-CD28 Abs, followed by cross-linking with goat anti-mouse IgG for 15 min at 37 °C. The lysates of CD4$^+$ T cells prepared from four healthy donors were pooled and immunoprecipitated with our in-house anti-human Zip6 Ab (hZIP6). Immunoprecipitates were analyzed by immunoblot analysis to detect Zap70.

Supplementary Figure 6

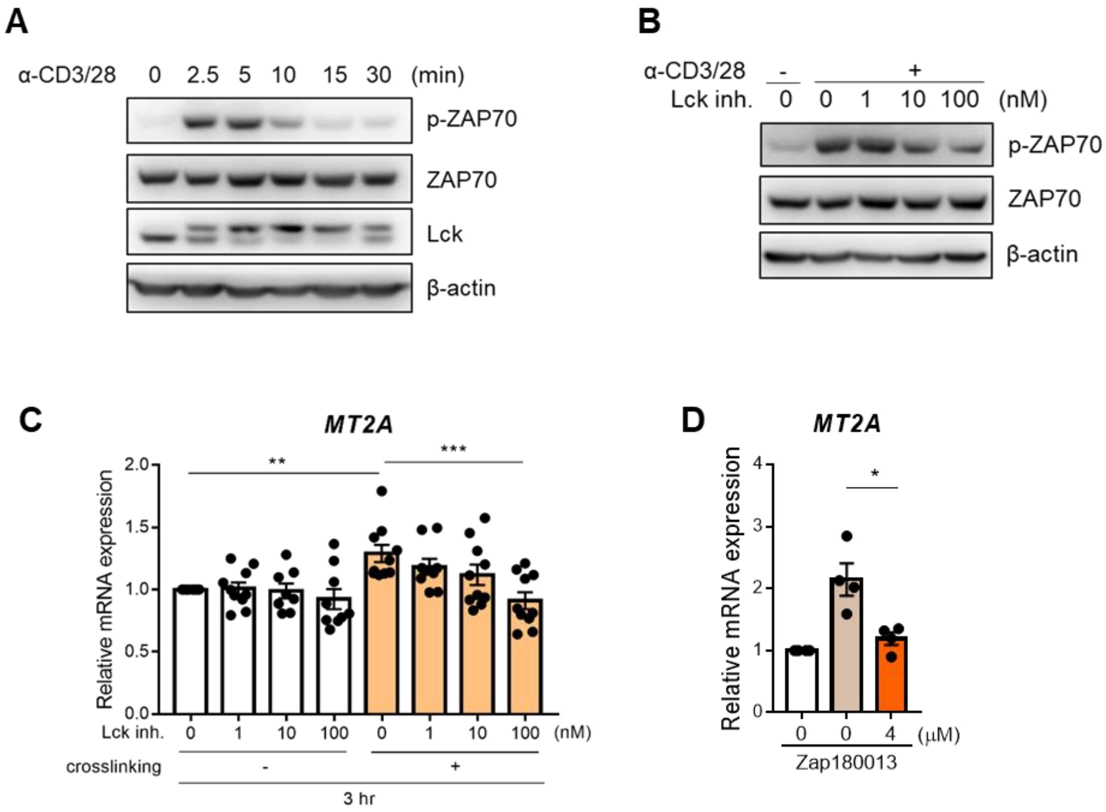

Figure 6. Effect of inhibition of early TCR signaling on MT2A expression.
(A) Jurkat cells were coated with anti-CD3 (2 μg/ml) and anti-CD28 (1 μg/ml) Abs, followed by crosslinking with goat anti-mouse IgG (2 μg/ml) for the indicated times at 37 °C. Cell lysates were prepared and immunoblotted for phosphorylated ZAP70 and Lck expression. **(B)** Jurkat cells were pre-treated with the indicated concentrations of Lck inhibitor followed by activation in the same manner as (A). **(C-D)** Purified $CD4^{+}$ T cells were pre-treated with Lck inhibitor or Zap70 inhibitor (Zap180013) for 30 min at 37°C, followed by coating with anti-CD3/28 Abs on ice, and cross-linking with anti-mouse IgG for 3 hr at 37°C. Metallothionein 2A (MT2A) mRNA was quantified by real-time RT-PCR. Bar graphs show the mean ± SEM. * = $p < 0.05$, ** = $p < 0.01$, and *** = $p < 0.005$ by two-tailed paired *t*-test.

Supplementary Figure 7

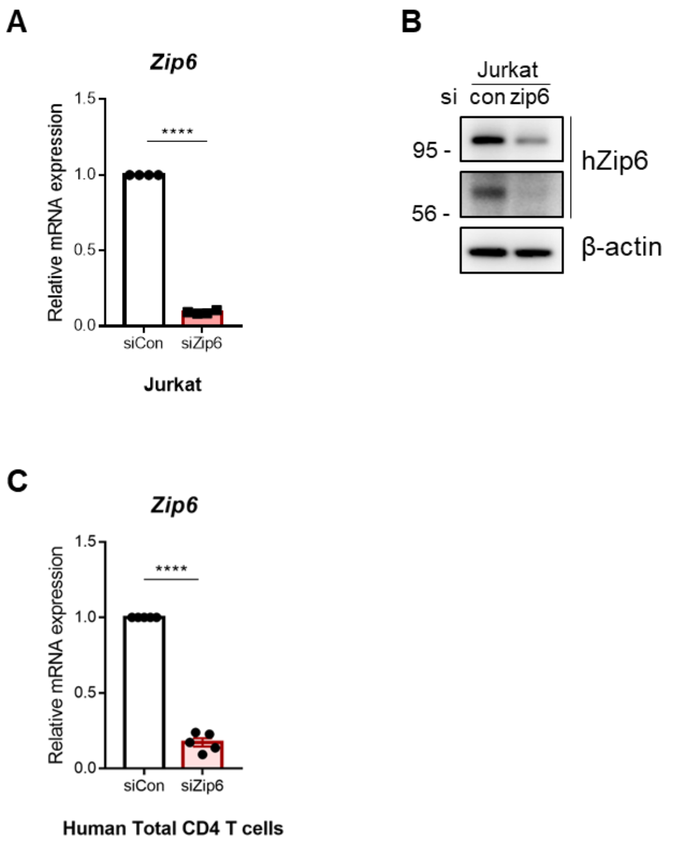

Figure 7. Zinc influx through ZIP6 in stimulated T cells
(A-C) Knockdown efficiency of Zip6. Jurkat cells (A-B) and primary CD4+ T cells (C) were transfected with human Zip6-specific or control siRNA (40 pM of both siRNAs). At 48 h post-transfection, the efficiency of Zip6-specific knockdown was evaluated by analysis of mRNA (A, C) and protein expression (B). Bar graphs show the mean ± SEM. **** = $p <$ 0.0001 by paired *t*-test.
